# Bi-directional regulation of acupuncture on extrahepatic biliary system: An approach in guinea pigs

**DOI:** 10.1038/s41598-017-14482-x

**Published:** 2017-10-25

**Authors:** Jingjun Zhao, Yutian Yu, Man Luo, Liang Li, Peijing Rong

**Affiliations:** 1 Institute of Acupuncture and Moxibustion, China Academy of Chinese Medical Sciences, Beijing, China; 2 Clinical Medical College of Acupuncture, Moxibustion and Rehabilitation, Guangzhou University of Chinese Medicine, Guangzhou, China; 3 Rudolf Boehm Institute of Pharmacology and Toxicology, Universität Leipzig, Härtelstrasse 16-18, 04107 Leipzig, Germany

## Abstract

Clinically, acupuncture affects the motility of the extrahepatic biliary tract, but the underlining mechanisms are still unknown. We applied manual acupuncture (MA) and electrical acupuncture (EA) separately at acupoints Tianshu (ST25), Qimen (LR14), Yanglingquan (GB34), and Yidan (CO11) in forty guinea pigs (4 groups) with or without atropinization under anesthesia while Sphincter of Oddi (SO) myoelectric activities and gallbladder pressure were monitored. In both MA and EA groups, stimulation at ST25 or LR14 significantly increased the frequency and amplitude of SO myoelectrical activities and simultaneously decreased the gallbladder pressure as compared to the pre-MA and pre-EA (*P* < 0.05). On the contrary, stimulation at GB34 or CO11 significantly decreased SO myoelectricity and increased the gallbladder pressure (*P* < 0.05). Pretreatment with atropine could abolish the effect of stimulation at acupoints ST25, GB34 and LR14 (*P* > 0.05), although significant myoelectricity increases were still inducible with MA or EA stimulation at CO11 (*P* < 0.05). In summary, acupuncture has bi-directional effects to gallbladder pressure and SO function, which probably due to autonomic reflex and somatovisceral interactions.

## Introduction

Acute and chronic hepatitis, hepatic cirrhosis, cholelithiasis, and other extrahepatic biliary diseases may be reflected as hypochondriac pain and jaundice. It has been confirmed by long-term clinical practice that acupuncture at ST25, LR14, GB34 as well as the auricular concha acupoint CO11 exerts good therapeutic effects on biliary diseases^1–3^. As medical technology and basic research progress, a growing number of evidences indicate that the motor dysfunction of the extrahepatic biliary system may be an important factor that induces biliary diseases^4–7^. The gallbladder is an organ that stores and concentrates bile and has rhythmic and tonic contractions. It is relaxing when the stomach is empty, but contracts after meals. The Sphincter of Oddi (SO) is a complex structure of smooth muscle almost entirely located within the duodenal wall wrapping around the end portion of the common bile duct, the pancreatic duct, and the vater ampulla. The gallbladder and SO are main objects for the observation of the extrahepatic biliary system since their activities are important under both physical and pathological conditions^8^.

In this study, we observed the effect of acupuncture at ST25, LR14, GB34 and CO11 on gallbladder pressure and SO motility in guinea pigs under physiological conditions by placing a small artificial bladder into the gallbladder and a double-hooked tungsten electrode through the SO. The purpose was to study the effect of acupuncture at ST25, LR14, GB34 and CO11 in regulating the motor function of the extrahepatic biliary system and factors that influence the effect, and thus to provide the physiological and experimental bases for the clinical practice of acupuncture.

## Results

### Effect of MA stimulation in normal guinea pigs

**Figure 1:**
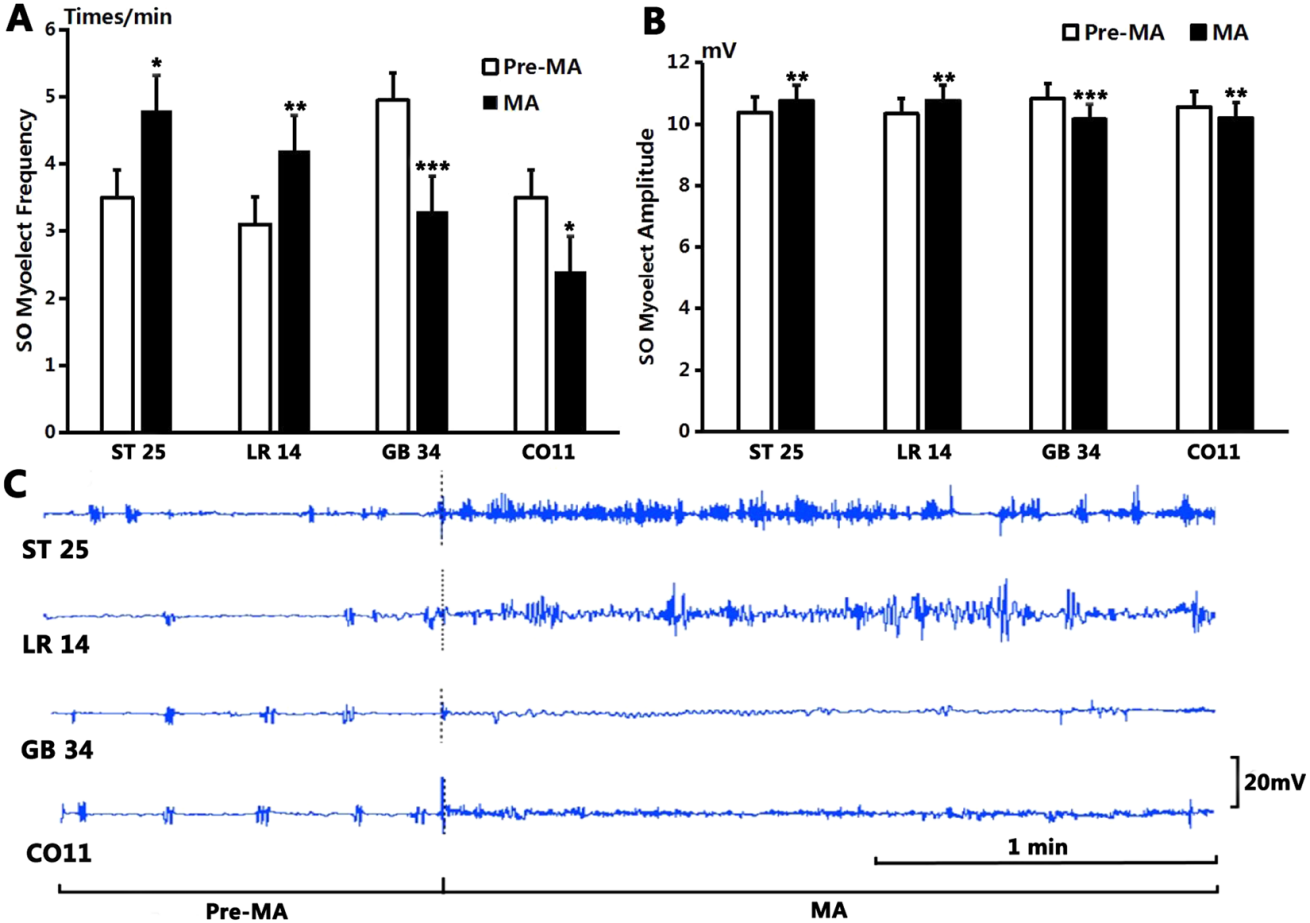
The effect on SO EMG frequency and amplitude by manual acupuncture. (**A**) The frequency of SO EMG increased significantly after stimulation at ST25 and continued for 1 min (*P* < 0.05) compared to that before MA; compared to the pre-MA level, SO EMG frequency increased significantly after stimulation at LR14 (*P* < 0.01), but decreased significantly after stimulation at GB34 (*P* < 0.001) and at CO11 (*P* < 0.05). (**B**) Compared to the benchmark level before MA, the amplitude of SO EMG increased significantly after stimulation at ST25 (*P* < 0.01) and at LR14 (*P* < 0.01), but decreased significantly after stimulation at GB34 (*P* < 0.001) and at CO11 (*P* < 0.01). Duration for MA stimulation was 1 min each time. (**C**) Sample traces of SO EMG show that the frequency and amplitude increased significantly after MA stimulation at ST25 and at LR14, but decreased significantly after stimulation at GB34 and at CO11.

As shown in Fig. 1, after MA stimulation at GB34, the average frequency of SO EMG decreased from the pre-stimulation level of 4.9 ± 0.23 times/min to 3.3 ± 0.37 times/min (*P* < 0.001, n = 10). The average amplitude decreased from the pre-MA level of 10.83 ± 0.08 mV to 10.16 ± 0.04 mV (*P* < 0.001, n = 10).

After stimulation at CO11, the average frequency of SO also decreased from the pre-MA level of 3.5 ± 0.31 times/min to 2.40 ± 0.27 times/min (*P* < 0.05, n = 10). The average amplitude decreased from the pre-MA level of 10.56 ± 0.07 mV to 10.24 ± 0.04 mV (*P* < 0.01, n = 10).

However, MA at LR14 increased the average frequency of SO EMG from 3.10 ± 0.35 times/min to 4.2 ± 0.36 times/min (*P* < 0.01, n = 10) and the average amplitude of SO electromyogram from 10.34 ± 0.04 mV to 10.77 ± 0.06 mV (*P* < 0.01, n = 10).

MA at ST25 increased the average frequency of SO EMG from 3.5 ± 0.31 times/min to 4.8 ± 0.36 times/min (*P* < 0.05, n = 10) and the average amplitude of SO EMG from 10.38 ± 0.04 mV to 10.76 ± 0.09 mV (*P* < 0.01, n = 10).

**Figure 2:**
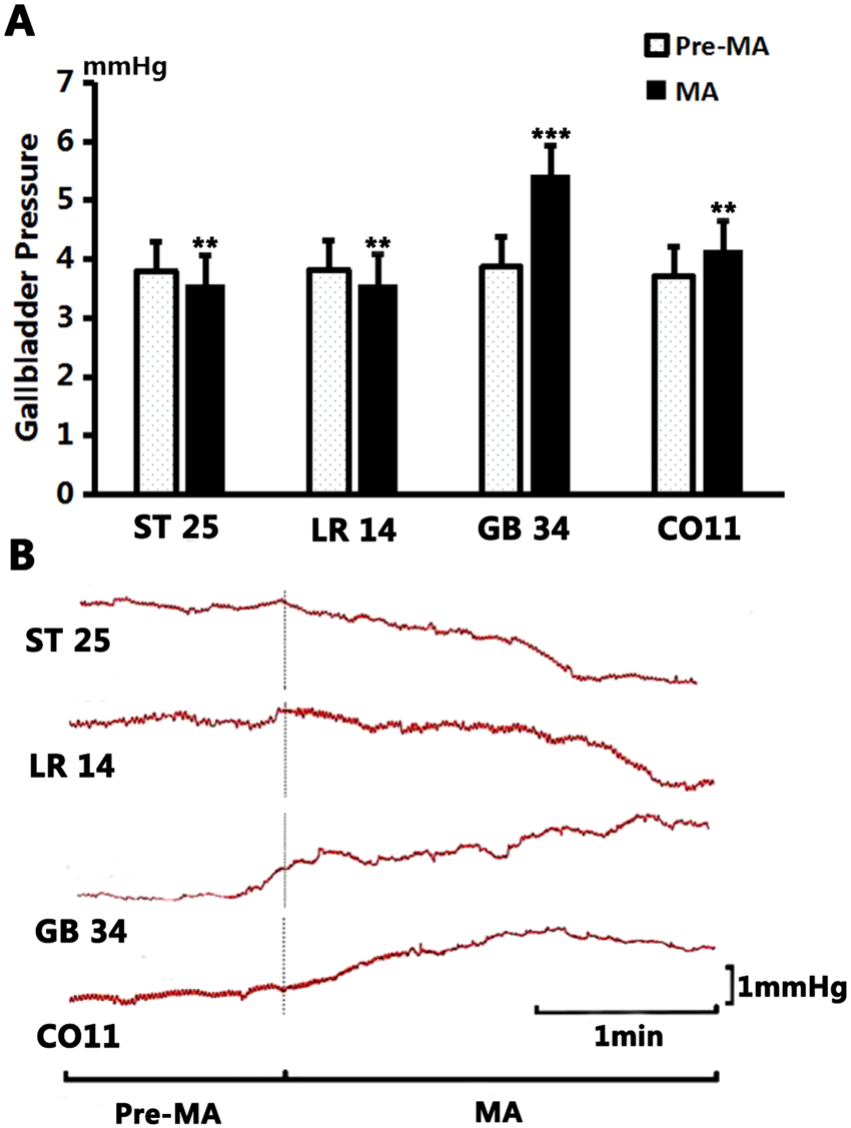
The changes of gallbladder pressure by manual acupuncture. (**A**) Compared to the benchmark level before MA, the gallbladder pressure decreased significantly after stimulation at ST25 (*P* < 0.01) and at LR14 (*P* < 0.01); and increased significantly after stimulation at GB34 (*P* < 0.001) and at CO11 (*P* < 0.01). Duration for MA stimulation was 1 min each time. (**B**) Sample traces of gallbladder pressure showing the effects of manual acupuncture at ST25, LR14, GB34 and CO11.

In terms of gallbladder pressure, as shown in Fig. 2, it increased from the pre-MA level of 3.87 ± 0.05 mmHg to 5.43 ± 0.12 mmHg (*P* < 0.001, n = 10) after stimulation at GB34. After stimulation at CO11, the gallbladder pressure increased from the pre-MA level of 3.71 ± 0.09 mmHg to 4.16 ± 0.09 mmHg (*P* < 0.01, n = 10). However, after MA at LR14, the gallbladder pressure decreased from 3.82 ± 0.05 mmHg to 3.58 ± 0.03 mmHg (*P* < 0.01, n = 10). After MA at ST25, the gallbladder pressure decreased from 3.8 ± 0.06 mmHg to 3.57 ± 0.05 mmHg (*P* < 0.01, n = 10).

### Effect of EA stimulation in normal guinea pigs

**Figure 3:**
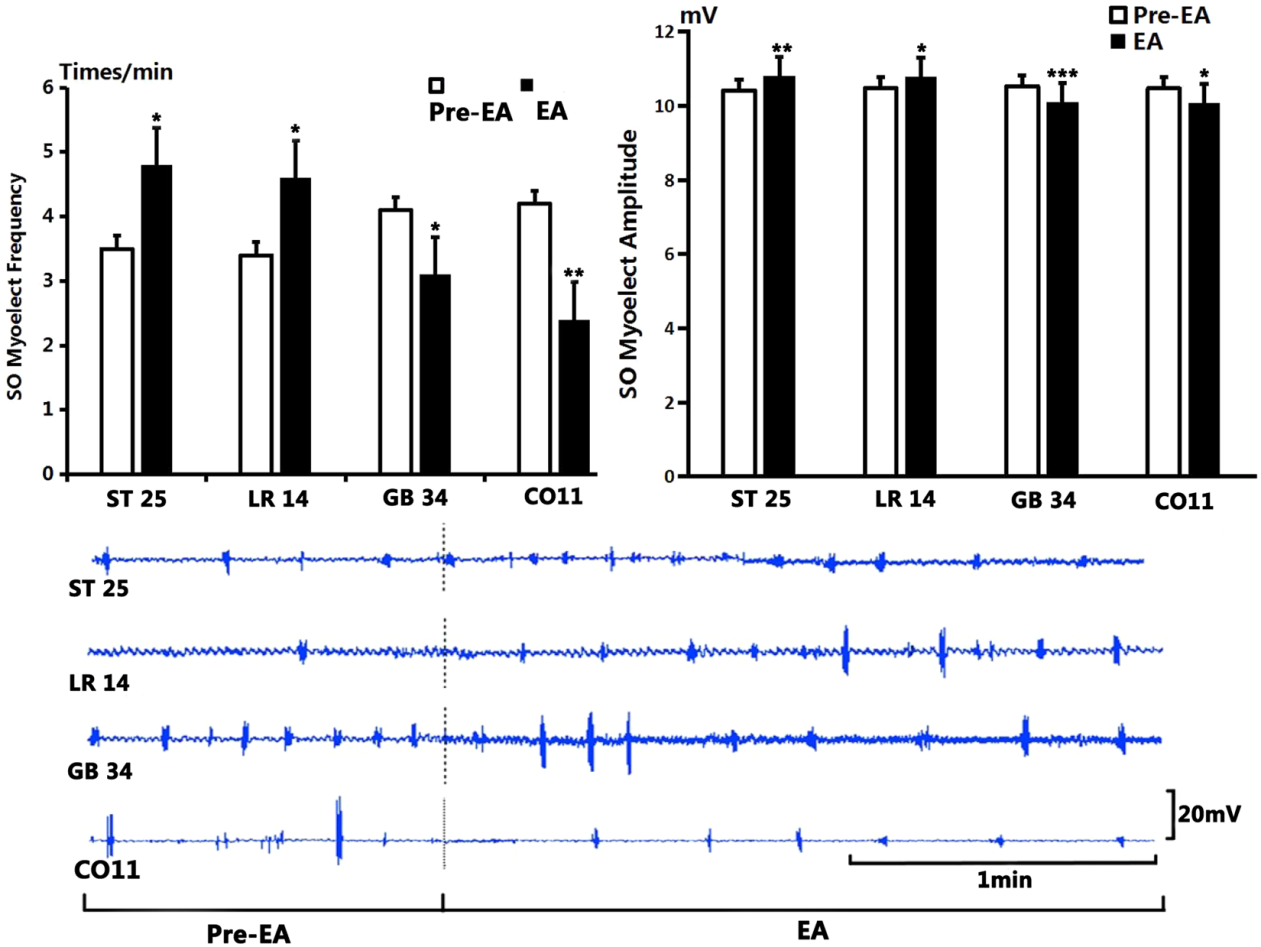
The effect on SO EMG frequency and amplitude by electroacupuncture. (**A**) Compared to the benchmark level before EA, the frequency of SO EMG increased significantly after stimulation at ST25 and at LR14 (*P* < 0.05); but decreased significantly after stimulation at GB34 (*P* < 0.05) and at CO11 (*P* < 0.01). (**B**) Compared to the benchmark level before EA, the amplitude of SO EMG increased significantly after stimulation at ST25 (*P* < 0.01) and at LR14 (*P* < 0.05) and decreased significantly after stimulation at GB34 (*P* < 0.001) and at CO11 (*P* < 0.05). (**C**): Sample traces of SO EMG after stimulation at ST25, LR14, GB34 and CO11.

As shown in Fig. 3, after stimulation at GB34, the average frequency of SO waves on EMG decreased from 4.1 ± 0.31 times/min to 3.1 ± 0.31 times/min (*P* < 0.05, n = 10). The average amplitude of SO wave decreased from 10.53 ± 0.06 mV to 10.11 ± 0.03 mV (*P* < 0.001, n = 10).

After EA at CO11, the average frequency of SO waves decreased from 4.2 ± 0.29 times/min to 2.4 ± 0.22 times/min (*P* < 0.01, n = 10). The average amplitude of SO waves decreased from 10.47 ± 0.08 mV to 10.08 ± 0.1 mV (*P* < 0.05, n = 10).

However, after EA at LR14, the average frequency of SO waves increased from 3.4 ± 0.27 times/min to 4.6 ± 0.31 times/min (*P* < 0.05, n = 10). The average amplitude of SO waves increased from 10.47 ± 0.06 mV to 10.79 ± 0.09 mV (*P* < 0.05, n = 10).

After EA at ST25, the average frequency of SO waves increased from 3.5 ± 0.31 times/min to 4.8 ± 0.25 times/min (*P* < 0.05, n = 10). The average amplitude of SO waves increased from 10.41 ± 0.06 mV to 10.81 ± 0.09 mV (*P* < 0.01, n = 10).

**Figure 4:**
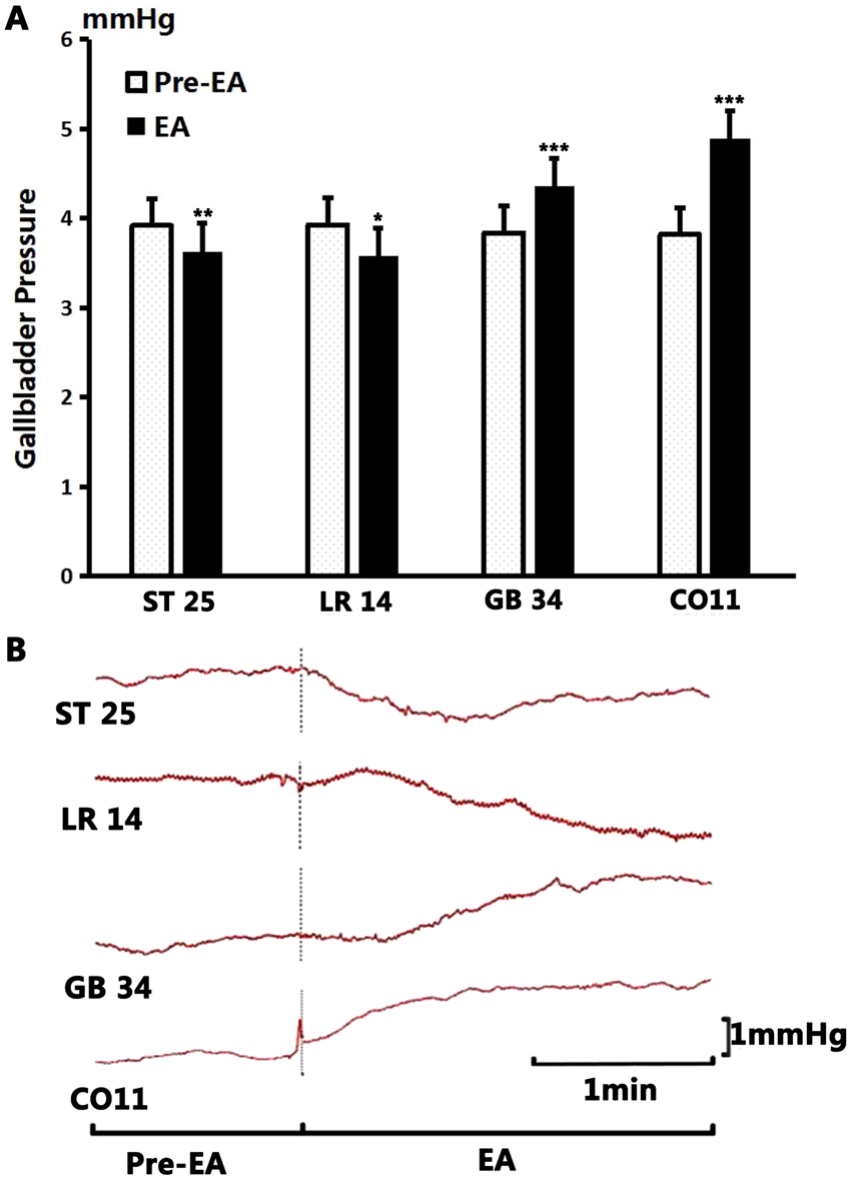
The change of gallbladder pressure by electroacupuncture. (**A**) Compared to the benchmark level before EA, the gallbladder pressure decreased significantly after stimulation at ST25 (*P* < 0.01), and at LR14 (*P* < 0.05); but increased significantly after stimulation at GB34 and at CO11 (*P* < 0.001). (**B**) Sample traces of gallbladder pressure changes after stimulation of different acupoints.

As shown in Fig. 4, after EA stimulation at GB34, the gallbladder pressure increased from the pre-EA level of 3.84 ± 0.06 mmHg to 4.36 ± 0.07 mmHg (*P* < 0.001, n = 10). After EA at CO11, the gallbladder pressure increased from 3.82 ± 0.07 mmHg to 4.39 ± 0.09 mmHg (*P* < 0.001, n = 10). However, after EA at LR14, the gallbladder pressure decreased from 3.93 ± 0.08 mmHg to 3.58 ± 0.05 mmHg (*P* < 0.05, n = 10). After EA at ST25, the gallbladder pressure also decreased from 3.92 ± 0.05 mmHg to 3.63 ± 0.09 mmHg (*P* < 0.01, n = 10).

There was no significant difference in the average frequency of SO waves between the MA group and the EA group at GB34, CO11, LR14 and ST25 (both *P* > 0.05) before stimulation in normal guinea pigs.

Before stimulation at GB34, CO11, LR14 and ST25 (both *P* > 0.05), no significant difference in the average amplitude of SO EMG was observed between the MA and EA groups in normal guinea pigs;

There was no significant difference in average gallbladder pressure between MA group and EA group at GB34, CO11, LR14 and ST25 (both P > 0.05) before stimulation in normal guinea pigs. No significant difference in the average frequency and amplitude of SO EMG was noted between the MA group and the EA group before or after stimulation at the four acupoints. As for the gallbladder pressure, only stimulation at CO11 produced a significant difference between the MA and EA group (MA: 5.43 ± 0.12, EA: 4.36 ± 0.07, *P* < 0.001, n = 10).

### Effect of MA stimulation in atropinized guinea pigs

**Figure 5:**
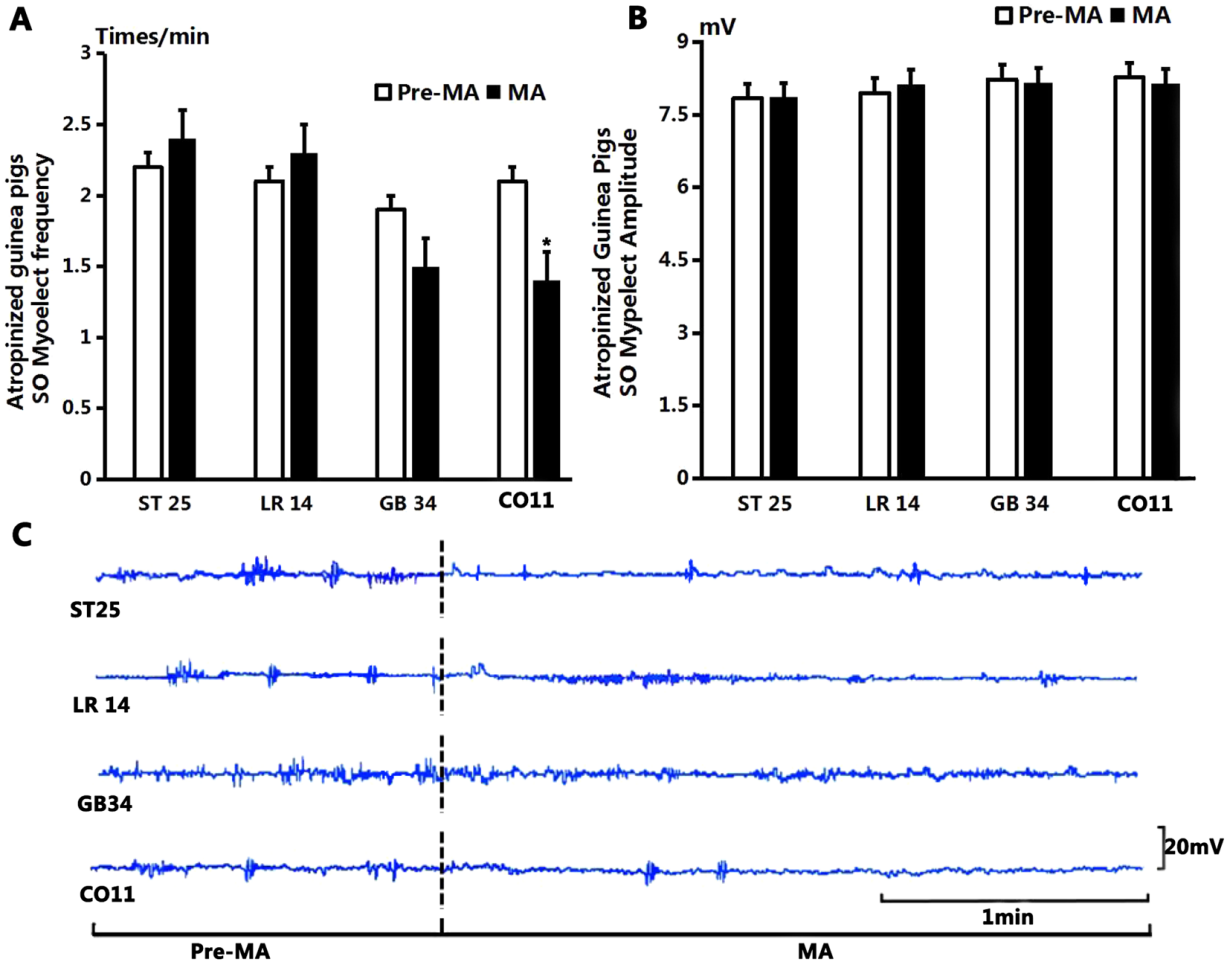
The effect on SO EMG frequency and amplitude of atropinized guinea pigs by manual acupuncture. (**A**) Compared to the control level before MA, the frequency of SO EMG had no significant change after stimulation at ST25, LR14 and GB34 (*P* > 0.05); but decreased significantly after stimulation at CO11 (*P* < 0.05). (**B**) Compared to the control level before MA, the amplitude of SO EMG had no significant difference after stimulation at ST25, LR14, GB34 and CO11 (*P* > 0.05). (**C**) Sample traces of SO EMG show that the frequency and amplitude of SO EMG had no obvious change after stimulation at both four acupoints.

As shown in Fig. 5, after stimulation at GB34, the average frequency of SO EMG decreased from 1.9 ± 1.75 times/min to 1.5 ± 1 times/min (*P* > 0.05, n = 10). The average amplitude of SO EMG decreased from 8.23 ± 0.27 mV to 8.17 ± 0.21 mV (*P* > 0.05, n = 10).

After MA at CO11, the average frequency of SO EMG decreased from 2.1 ± 0.31 times/min to 1.4 ± 0.22 times/min (*P* < 0.05, n = 10). The average amplitude of SO EMG decreased from 8.27 ± 0.04 mV to 8.14 ± 0.19 mV (*P* > 0.05, n = 10).

After MA at LR14, the average frequency of SO EMG increased from 2.1 ± 0.18 times/min to 2.3 ± 0.3 times/min (*P* > 0.05, n = 10). The average amplitude of SO EMG increased from 7.95 ± 0.07 mV to 8.13 ± 0.15 mV(*P* > 0.05, n = 10).

After MA at ST25 the average frequency of SO EMG increased from 2.1 ± 0.28 times/min to 2.4 ± 0.31 times/min (*P* > 0.05, n = 10). The average amplitude of SO EMG increased from 7.84 ± 0.08 mV to 7.86 ± 0.07 mV (*P* > 0.05, n = 10).

**Figure 6:**
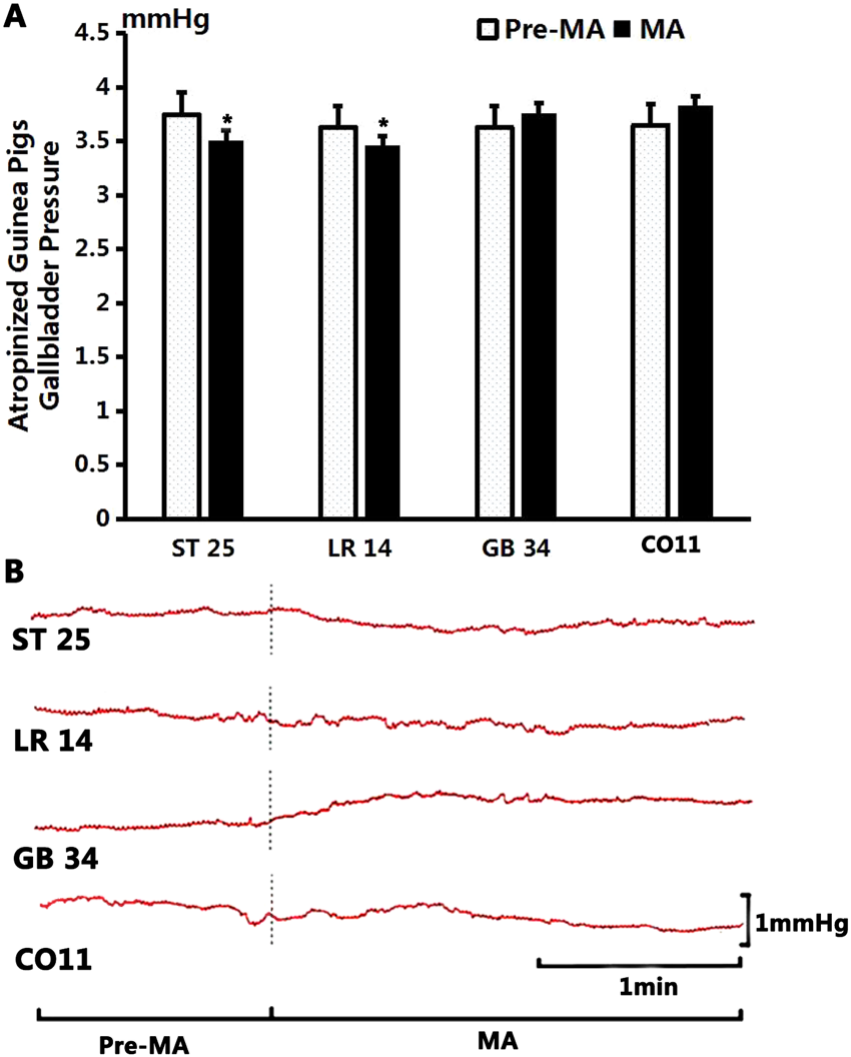
The effect of manual acupuncture on gallbladder pressure in atropinized guinea pigs. (**A**) Compared to the benchmark level before MA, the gallbladder pressure decreased significantly after MA stimulation at ST25 (*P* < 0.05) and at LR14 (*P* < 0.05); the gallbladder pressure showed no significant change after stimulation at GB34 (*P* > 0.05) and at CO11 (*P* > 0.05). (**B**) Sample traces of gallbladder pressure changes after stimulation of different acupoints.

As shown in Fig. 6, after stimulation at GB34, the gallbladder pressure increased from the pre-MA level of 3.63 ± 0.88 mmHg to 3.76 ± 0.96 mmHg (*P* > 0.05, n = 10). After MA at CO11, the gallbladder pressure increased from 3.65 ± 0.07 mmHg to 3.83 ± 0.11 mmHg (*P* > 0.05, n = 10). After MA at LR14, the gallbladder pressure decreased from 3.63 ± 0.07 mmHg to 3.46 ± 0.02 mmHg (*P* < 0.05, n = 10). After MA at ST25 the gallbladder pressure decreased from 3.75 ± 0.07 mmHg to 3.51 ± 0.07 mmHg (*P* < 0.05, n = 10).

### Effect of EA stimulation in atropinized guinea pigs

**Figure 7:**
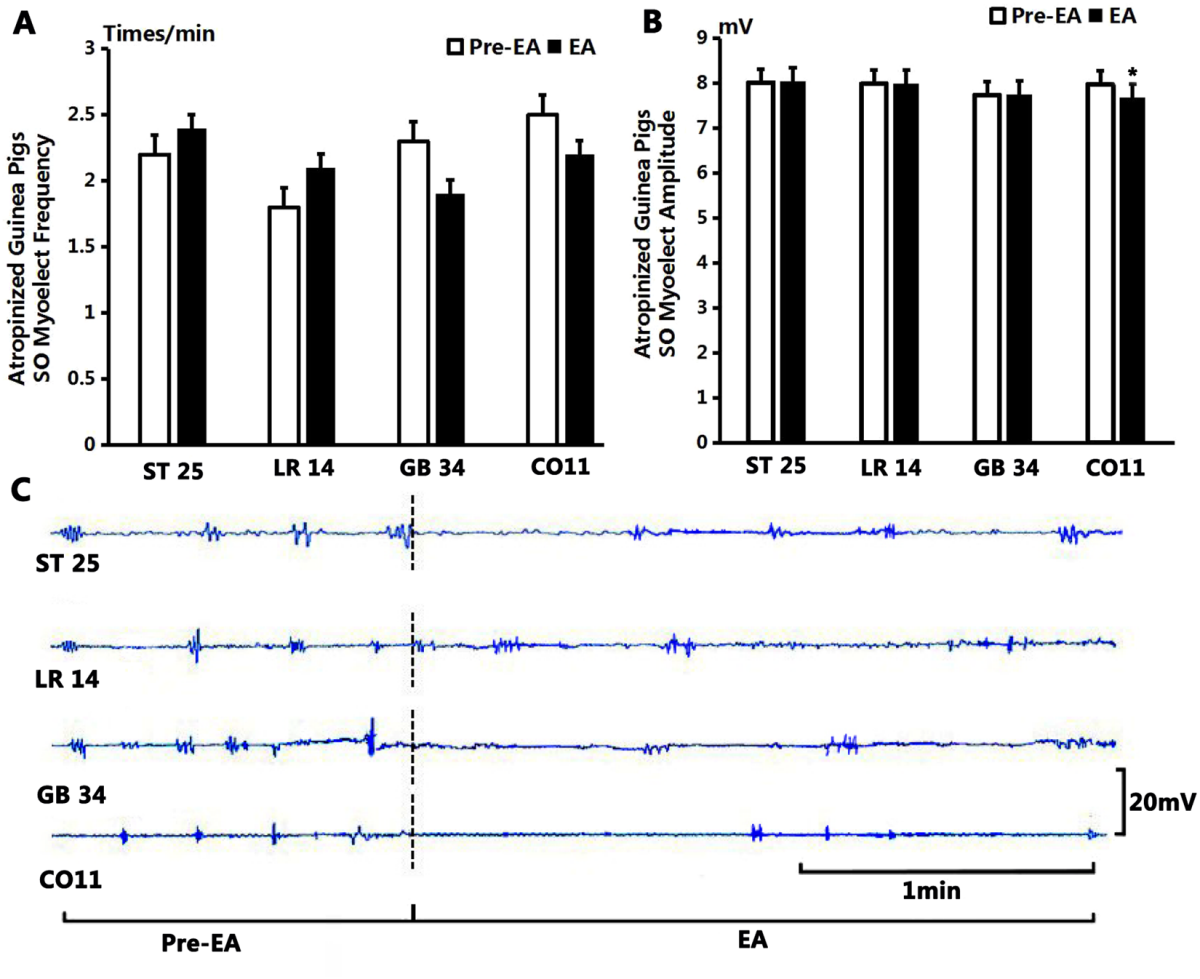
The effect on SO EMG frequency and amplitude by electroacupuncture. (**A**) Compared to the benchmark level before EA, the frequency of SO EMG had no significant difference after stimulation at ST25, LR14, GB34 and CO11 (*P* > 0.05). (**B**) Compared to the benchmark level before EA, the amplitude of SO EMG had no significant difference after stimulation at ST25, LR14 and GB34 (*P* > 0.05); but decreased significantly after stimulation at CO11 (*P* < 0.05). (**C**) Sample traces of SO EMG show that the frequency and amplitude had no obvious change after EA at ST25, LR14 and GB34, but the amplitude had a significant decrease after EA at CO11.

As shown in Fig. 7, after stimulation at GB34, the average frequency of SO EMG decreased from the pre-EA level of 2.3 ± 0.21 times/min to 1.9 ± 0.23 times/min (*P* > 0.05, n = 10). The average amplitude of SO EMG decreased from 7.74 ± 0.08 mV to 7.75 ± 0.09 mV (*P* > 0.05, n = 10).

After EA at CO11, the average frequency of SO EMG decreased from 2.5 ± 0.27 times/min to 2.2 ± 0.33 times/min (*P* > 0.05, n = 10). The average amplitude of SO EMG decreased from 7.98 ± 0.09 mV to 7.68 ± 0.09 mV (*P* < 0.05, n = 10).

After EA at LR14, the average frequency of SO EMG increased from 1.8 ± 0.29 times/min to 2.1 ± 0.31 times/min (*P* > 0.05, n = 10) and the average amplitude of SO EMG increased from 7.99 ± 0.06 mV to 8 ± 0.13 mV (*P* > 0.05, n = 10). After EA at ST25 the average frequency of SO EMG increased from 2.2 ± 0.2 times/min to 2.4 ± 1.08 times/min (*P* > 0.05, n = 10). The average amplitude of SO EMG increased from 8.02 ± 0.085 mV to 8.05 ± 0.1 mV (*P* > 0.05, n = 10).

**Figure 8:**
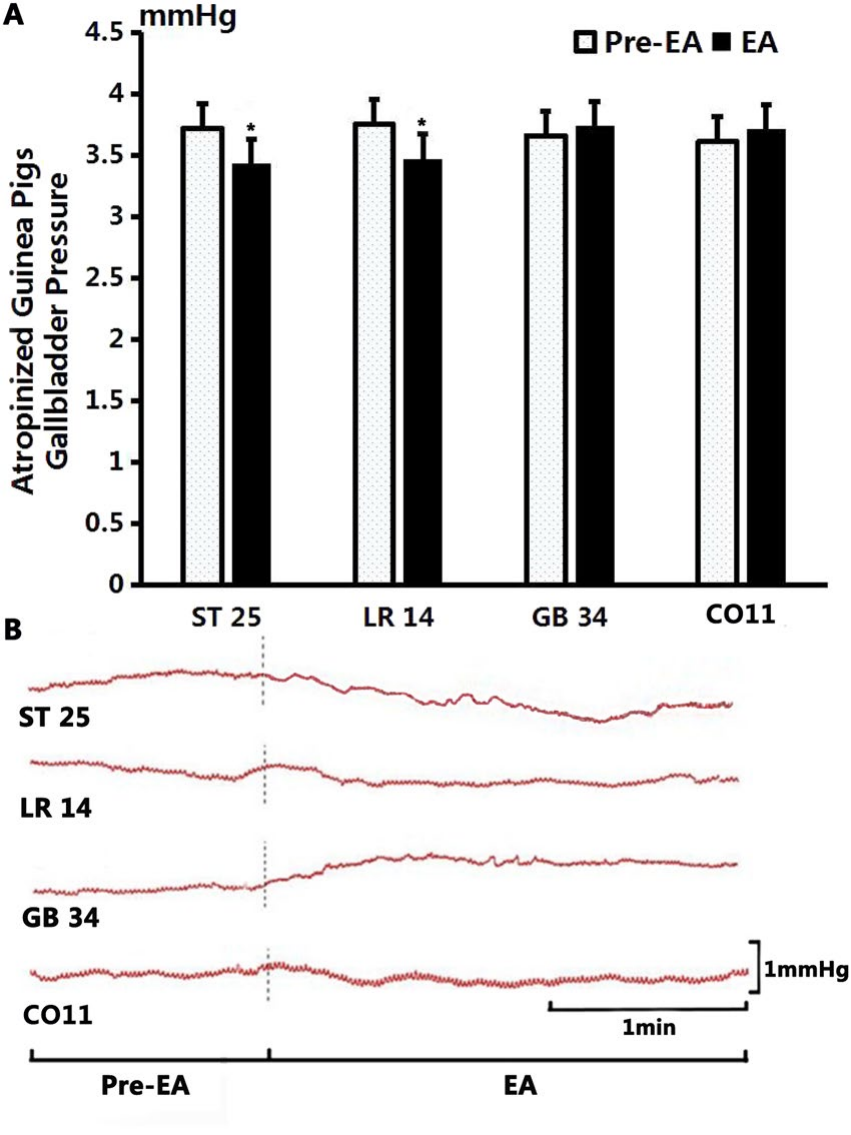
The effect of electroacupuncture on gallbladder pressure in atropinized guinea pigs. (**A**) Compared to the benchmark level before EA, the gallbladder pressure showed a significant change after EA stimulation at ST25 (*P* < 0.05) and at LR14 (*P* < 0.05); but no obvious change was noted after stimulation at GB34 (*P* > 0.05) and at CO11 (*P* > 0.05). (**B**) Sample traces of gallbladder pressure changes after stimulation of different acupoints.

As shown in Fig. 8, after stimulation at GB34, the gallbladder pressure increased from 3.66 ± 0.12 mmHg to 3.74 ± 0.09 mmHg (*P* > 0.05, n = 10). After EA at CO11, the gallbladder pressure increased from 3.61 ± 0.1 mmHg to 3.71 ± 0.1 mmHg (*P* > 0.05, n = 10). After EA at LR14, the gallbladder pressure decreased from 3.75 ± 0.05 mmHg to 3.47 ± 0.07 mmHg (*P* < 0.05, n = 10). After EA at ST25, the gallbladder pressure decreased from 3.72 ± 0.09 mmHg to 3.43 ± 0.07 mmHg (*P* < 0.05, n = 10).

There was no significant difference of the average frequency and amplitude of SO EMG at the acupoints of GB34, CO11, LR14 and ST25 before or after stimulation between the MA group and the EA group in atropine guinea pigs, indicating the effect of MA and EA stimulation is interchangeable.

## Discussion

In summary, stimulation at ST25 and LR14 significantly increased the frequency and amplitude of SO myoelectrical activities and decreased the gallbladder pressure as compared to the pre-MA and pre-EA level. In contrast, stimulation at GB34 and CO11 caused a significant decrease of the SO myoelectricity and an increase of the gallbladder pressure. Pretreatment with atropine inhibited the effect of stimulation at acupoints ST25, GB34 and LR14, but significant myoelectricity increase was still noted after MA or EA stimulation at CO11.

This bi-directional effect of acupuncture on the extrahepatic biliary system was observed between acupuncture points ST25/LR14 and GB34/CO11. And this interesting paradox is obviously related to the sympathetic/parasympathetic nervous regulation of acupuncture, which agrees with the viewpoint of traditional Chinese medicine. Stimulation at ST25 and LR14 excites the cholecystic parasympathetic nervous system, resulting in decreased gallbladder pressure, while stimulation at GB34 or CO11 activates the cholecystic sympathetic nervous system and produces an increase of the gallbladder pressure. Atropine inhibits the effect of stimulation at acupoints ST25, GB34 and LR14, but cannot abolish the effect of stimulation at the auricular acupoint CO11.

Studies have shown that the reactivity to agonists CCK-8 of rabbits with chronic cholecystitis significantly decreased, and myoelectric activity of SO and its response to agonists CCK-8 and KCI are decreased in rabbits with chronic cholangitis both *in vivo* and *in-vitro* ^9^. Both clinical and experimental types of research have confirmed that acupuncture at GB34, LR14, and auricular concha has a good curative effect on corresponding biliary diseases^10–13^.

GB34 is an acupoint of the foot Shaoyang gallbladder Meridian, and acupuncture at GB34 may improve the function of the liver and the gallbladder^3,14^. Treatment of biliary diseases by acupuncture at GB34 has a long history and can be traced to the ancient medical book of ***Inner Canon of the Yellow Emperor*** ^15^.

LR14 is a front-mu point of the Liver Meridian. It is also a key point where the Spleen Meridian, the Liver Meridian of Foot-Jueyin and Yinwei Meridian converge and cross. Acupuncture at LR14 can effectively promote the function of the biliary system and has a good curative effect on biliary and liver diseases^16,17^. Current treatment methods for SO dysfunction include calcium channel blockers, smooth muscle relaxant and Oddi sphincterotomy^18^. However, not all patients with gallbladder motor dysfunction benefit from surgical interventions^19,20^.

Modern researches have shown that the vagus nerve is involved in the regulation of the gallbladder and the motor function of SO^21–23^. The auricular concha acupoint CO11 used in this study is located in cymba conchae, an area where the auricular branch of the vagus nerve concentrates^24,25^. Interestingly the auricular branch of the vagus nerve is the only branch of the vagus nerve that distributes at the superficial part of the human body^26^. Auricular acupuncture (AA) at CO11 used herewith could stimulate the vagus nerve directly.

Venous injection of atropine has an inhibitory effect on SO EMG of guinea pigs. EA or MA stimulation at CO11 has an excitatory effect on SO contraction of guinea pigs. It indicates that acupuncture at CO11 can partially block the inhibitory effect of atropine on SO EMG. Though the exact pathway for this effect remains unclear, it may be partially attributed to the secretion of cholecystokinin (CCK) as an important ghrelin that has an excitatory effect on the SO EMG^27^.

The afferent input of acupuncture may affect the gallbladder and SO of the extrahepatic biliary system mainly through the two pathways of somatic nerves and vessel wall plexus, and then to the CNS to trigger the flexural responses of corresponding tissues via the autonomic nervous system (ANS). The vagus nerve maintains the tension of the gallbladder and controls the dynamic contractions of the gallbladder in digestion. This regulation is controlled by the thyrotropin-releasing hormone secreted by the dorsal nucleus of vagus nerves through the vagus nerve and peripheral M receptors^28^. The vagus nerve is also involved in coordinating the SO and the gallbladder during digestion^29^. The emptying ability of the gallbladder is compromised and the risk of cholelithiasis rises when the vagus nerve is cut^30^. The dorsal raphe nucleus is involved in the regulation of gallbladder through the vagus nerve and the sympathetic nerve^31^.

The nervous regulation of the extrahepatic biliary system motility is performed via the sympathetic nerve in coordination with the vagus nerve^32^. Sympathetic nerve mediates the regulation of gallbladder by the ventrolateral medulla oblongata^33^. Sympathetic nerve maintains the tension of the gallbladder during digestion and activates the alpha, beta adrenergic receptors in the gallbladder. The gallbladder is in an excited state when alpha receptors are activated, but in an inhibitory state when the beta-adrenergic receptors are activated. Since stimulation of the vagus nerve has an excitatory effect on the gallbladder, the vagus nerve and the sympathetic nerve may co-regulate the contraction and relaxation of the gallbladder and the SO and promote the secretion of bile.

Sympathetic neurons that dominate the motility of the gallbladder are mainly distributed at the lateral horn of the T_4_-T_10_ spinal cord. Parasympathetic neurons that dominate the motility of the gallbladder are mainly distributed in the dorsal nucleus of the vagus nerve in the medulla oblongata. Sympathetic neurons that control the SO are mainly distributed at the lateral horn of the T_6_-T_12_ spinal cord and parasympathetic neurons that control the SO are mainly distributed in the dorsal nucleus of the vagus nerve in the medulla oblongata. Since somatic afferent signals from ST25 mainly project to the T_9_-T_11_, LR14 to T_3_-T_6,_ GB34 to L_4_-L_5_ spinal cord, there is quite an overlap of the distribution of spinal sympathetic neurons that control the motility of the extrahepatic biliary system^34^. Results of this study have shown that after stimulation at ST25 and LR14, the frequency and amplitude of SO EMG increased significantly and the gallbladder pressure decreased compared to the pre-acupuncture levels. Acupuncture at these acupoints may activate the sympathetic tone controlling the biliary motility, creating an overall inhibitory effect to relax the gallbladder. There could also be other mechanisms, of course. For example, nitric oxide also participates in the regulation of SO contractions^35^. The effect of acupuncture on these potential factors remains to be studied.

Limit of this experiment is that the tests were made only under the physiological conditions of guinea pigs, knowing that biliary diseases may alter the reactivities of the system. Further studies shall be made to test the effect of acupuncture on different pathological models such as cholesterol gallstone disease and chronic cholecystitis, or to test the effect of simultaneous stimulation at the body and auricular acupoints on the motility of SO and the gallbladder pressure. In addition, more species need to be experimented to confirm these findings, particularly in non-human primates which are much closer to humans. And in-depth mechanisms also need to be explored. Such studies will help to unveil the relations between the regulatory mechanisms of acupuncture and the location of acupoints, providing a more sound experimental basis to the clinical practice of acupuncture treatment for extrahepatic biliary diseases, which may reduce the side effects of surgical interventions.

Another limitation needs to be noted. Acupoints are usually used in combination with each other. Studying each acupoint’s effect is appropriate to elicit specific physiologic responses, but this is rarely the case in clinical use. Since the acupoints are not stimulated as a set, it is impossible to extrapolate the individual effect of acupoints to the set, mainly due to the paradoxical effect of the pairs of acupoints.

## Methods

Forty adult guinea pigs of both sexes with body weights ranging from 350 g to 400 g were provided by the Beijing Xishan Farm [The production license is SCXF-(Beijing)-2011–2011]. After being purchased, the animals were habituated to the experimental environment for one week during which they were fed with standard forage. To guarantee enough intakes of vitamins, each guinea pig was given carrot of half of its body weight besides standard forage daily. All animals had free access to water and food and were exposed to alternating 12-hr light and darkness. The room temperature was set between 23 °C and 25 °C. This animal experiment was approved by CACMS Animal Ethics Committee. All experimental protocols were performed strictly followed the *Guideline on the Humane Care and Use of Laboratory Animals* issued by the Ministry of Science and Technology of the People’s Republic of China in 2006.

On the day of the experiment all animals were anesthetized with an intraperitoneal injection of 10% urethane (1.2 ml/100 g). The animals’ body temperature was kept at (37.5 ± 0.2)°C during the experiment. Corneal reflex and leg withdrawal reflex were checked from time to time and 0.5 ml of the anesthetic was added each time to keep animals in a stable anesthetic state.

Materials and equipment used in the experiment included Powerlab S/4 data acquisition system (Ed Instrument of Shanghai International Trade Co., Ltd.), General Purpose Amplifier (Ed Instrument of Shanghai International Trade Co., Ltd.), H-KWDY-III animal thermostatic control system (Shanghai Alcott Biotech Co., Ltd.), acupuncture needles (0.35 mm × 10 mm, Suzhou Hua Tuo Medical Instruments Co., Ltd.), platinum-wire electrodes (0.25 mm, World Precision Instruments, Inc), MLT1199 pressure transducer (AD Instruments, Inc), artificial pressure capsules (1 mm, homemade), soft rubber catheters (0.5 mm × 20 cm) and two T connection heads.

## Animal preparation

The forty adult guinea pigs of both sexes were randomly divided into four groups by random numerical table method. In the manual-acupuncture (MA) normal group and the electroacupuncture (EA) normal group, bilateral ST25, LR14, GB34, and CO11 (an auricular acupoint where auricular vagus nerve concentrates) were stimulated by MA and EA respectively. In the MA atropinization group and EA atropinization group, atropine in saline (0.25 mg/kg) was dripped into the great saphenous vein of guinea pigs with a dose of 10 mL/kg and a speed of 20 drops/min with MA or EA stimulation simultaneously applied at bilateral ST25, LR14, GB34, or CO11.

## Experimental procedures

After 12 hours of fasting with free access to water, guinea pigs were anesthetized with an injection of 100 g/mL urethane at the abdomen and then fixed on constant temperature heating plates in supine position. The temperature was set at 37 ± 0.5 °C. The fur on the abdomen and GB34 point was removed and a 10-mm longitudinal incision was made along the ventral midline from the xiphoid process to expose the gallbladder, the duodenum and the common bile duct. Gallbladder intubation: a 12-mm incision was made at the bottom of the gallbladder and an artificial bladder filled with saline was placed into the gallbladder before the incision was closed. A soft rubber tube connected the artificial bladder with the pressure transducer (MLT1199) and the pressure transducer was plugged into the General Purpose Biological Signal Amplifier. Gallbladder pressure signals were input into the PowerLab S/4 data acquisition system. A double-hooked tungsten electrode was placed in the SO and the electrode wires were placed in parallel to the longitudinal axis of the duodenum. The other end of the electrode was connected to the General Purpose Amplifier. Signals were input into the PowerLab S/4 data acquisition system.

After the completion of the operation, guinea pigs were given a celiac injection of 5 ml of liquid paraffin to keep the tissue moist and the incision was covered by a cotton ball infiltrated with liquid paraffin to keep the tissue fresh and moist. After the operation, the EMG of SO and gallbladder pressure of guinea pigs under physiological condition were recorded for 30 min. Acupuncture operation started when stable electrical and pressure curves were recorded, and all MA and EA operations were performed in a fixed order from CO11, LR14, ST25 to GB34.

## Acupuncture

### Acupoint positioning

Acupoints were positioned with reference to the Commonly-used Acupuncture Points for Laboratory Animals^26^ and the positioning of acupoints on the human body. ST25 of guinea pigs was positioned on the middle abdomen, 0.5 cm lateral to the center of the umbilicus. LR14 of guinea pigs was positioned at the 6^th^ intercostal space. GB34 of guinea pigs was at the posterolateral cornor of the knee joint and 0.3 mm underneath the fibula capitulum. CO11 was at the cavity of the concha.

### Manual-acupuncture methods

To investigate whether acupuncture at heterotopic acupoints affected SO EMG activity in normal guinea pigs, in our experiment manual acupuncture (MA) was applied separately at ST25, LR14, GB34, and CO11.

ST25 of guinea pigs was positioned on the middle abdomen, 0.5 cm lateral to the center of the umbilicus. Acupuncture needle was inserted through ST25 perpendicularly to the depth of 0.2 cm and then twisted with the technique of mild reinforcing and attenuating at a frequency of ~160 times/min for 1 min.

LR14 was positioned at the 6^th^ intercostal space. Acupuncture needle was inserted through LR14 perpendicularly to the depth of 0.2 cm and then twisted with the technique of mild reinforcing and attenuating at a frequency of ~160 times/min for 1 min.

GB34 was at 0.4 cm upper the lateral side of Housanli (the posterolateral corner of the knee joint and 0.3 mm underneath the fibula capitulum). Acupuncture needle was inserted through GB34 perpendicularly to the depth of 0.2 cm and then twisted with the technique of mild reinforcing and attenuating at a frequency of ~160 times/min for 1 min.

CO11 was positioned at the cavity of the concha. Acupuncture needle was inserted through CO11 perpendicularly to the depth of 0.1 cm and then twisted with the technique of mild reinforcing and attenuating at a frequency of ~160 times/min for 1 min.

### EA methods

We punctured one needle in the acupoint with another one 3 mm near the acupoint. The needle on the acupoint was connected to the positive electrode, and the other needle was connected to the negative electrode. G6805-I electro-acupuncture therapeutic apparatus (Shanghai Medical Electronic Instrument Factory) was used in the experiment and parameters were set as follows: the frequency of condensation wave = 50 Hz, the frequency of rarefaction wave = 4 Hz, pulse width = 0.5 ms, output voltage = 2 V, output current = 1 mA, and the peak-to-peak amplitude = 0~60 (1 kΩ load).

### Measurement and analysis method

**Figure 9:**
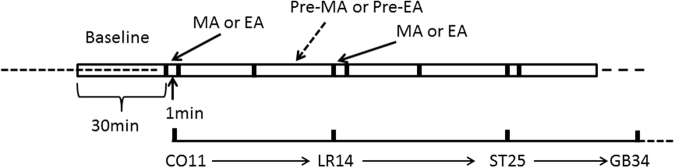
Measurement Procedure.

The slow wave of SO EMG of guinea pigs showed cyclical and rhythmic changes after 12 hours of fasting. It could generally be divided into a high-activity phase and a low-activity phase. The fast wave of SO EMG was bi-directional asymmetric needle wave and mainly took the form of peak clusters that often appeared at slow wave platform. The amplitude and frequency of the fast wave and the high activity phase of slow wave were recorded and analyzed in the experiment: (1) the basic average amplitude of the high-activity phase of slow wave was calculated by averaging the amplitudes in 30 min and the basic average frequency of the high-activity phase of slow wave was calculated by averaging the frequencies of the corresponding sections (times/min) before stimulated by EA or MA every time. The data of MA or EA recorded by stimulated at these acupoints sequence and every acupoint was stimulated for 1 min and after every time the stimulus was stopped for five minutes until the next Pre-MA or Pre-EA recorded began (Fig. 9), repeat the measurement 10 times by the stimulus sequence and then analyze the data. The post-stimulation average amplitude of the high-activity phase of slow wave was calculated by averaging the amplitudes in 1 min each (mV/cm) and the average frequency of the high-activity phase of slow wave was calculated by averaging the frequencies of the corresponding sections (times/min) after EA or MA every time; (2) the basic gallbladder pressure was calculated by averaging the amplitudes in 30 min (mmHg) before stimulation and the post-stimulation gallbladder pressure was calculated by averaging the amplitudes in 1 min each (mmHg) after MA or EA at these acupoints sequentially.

The reading of equipment was returned to zero before the experiment started. The sampling frequency of Chart 5.0 recording system was 200 Hz. The low-pass frequency for online SO EMG record was 10 Hz, 1 Hz for the offline digital filter, 2 Hz for gallbladder pressure recording and 1 Hz for the offline digital filter.

### Statistical analysis

All data were analyzed online and offline using the Mac Lab system. SO EMG and gallbladder pressure were recorded continuously prior to and during acupuncture stimulation. We analyzed the frequency (times/min) and amplitude (mV) of SO EMG and the gallbladder contraction wave. Statistical analysis was performed using Sigma plot 12.5 software (Systat, United States). All data were shown as the mean ± SE. Oneway RM ANOVA was used to test the statistical significance of the difference of means of all groups of data sets had a normal distribution. *P* < 0.05 was considered statistically significant.
